# The importance of discovery science in the development of therapies for the critically ill

**DOI:** 10.1186/s40635-020-00304-4

**Published:** 2020-05-26

**Authors:** Nicole P. Juffermans, Peter Radermacher, John G. Laffey, on behalf of the Translational Biology Group

**Affiliations:** 1 Laboratory of Experimental Intensive Care and Anesthesiology, AmsterdamUMC, location AMC, Amsterdam, the Netherlands; 2 OLVG Hospital, Amsterdam, the Netherlands; 3 Anaesthesia, School of Medicine, National University of Ireland, Galway, Ireland; 4 Regenerative Medicine Institute (REMEDI) at CÚRAM Centre for Research in Medical Devices, Biomedical Sciences Building, National University of Ireland, Galway, Ireland

**Keywords:** Basic science, Translational science, Discovery science, Critical care

## Abstract

Discovery science, a term which encompasses basic, translational, and computational science with the aim to discover new therapies, has advanced critical care. By combining knowledge on inflammatory and genomic pathways with computational methods, discovery science is currently enabling us to optimize clinical trials design by predictive enrichment and to move into the era of personalized medicine for complex syndromes such as sepsis and ARDS. Whereas computational methods are gaining in interest, efforts to invest in basic and translational science in critical care are declining. As basic and translational science is essential to advance our understanding of the pathophysiology of organ failure, this loss of interest may result in failure to discover new therapies for the critically ill. A renewed emphasis on basic and translational science is essential to find solutions for fundamental questions that remain in critical care. This requires a strategy to prioritize basic and translational science as an essential component within the critical care research “toolkit.” Key aspects of this strategy include an increased focus on basic science in critical care medical curricula as well as in critical care platforms such as conferences and medical journals. Training of critical care clinician scientists in basic and translational research will require new organizational models within the academic institutions, as well as the development of new funding opportunities for early career critical care clinician scientists.

## Background

Advances in discovery science, which encompasses basic, translational, and computational research, underlie key discoveries and new therapies in medicine, and constitute the “pipeline” for leading edge medical advances. These advances have revolutionized medical care, leading to numerous discoveries that have increased our life span and our productivity, with a decrease in health care-related costs. Thereby, return on investment in discovery science has been strikingly high [1]. In medicine, basic science usually refers to research that is not directly related to therapeutic strategies, whereas translational science refers to the translation of findings in basic science to the development of potential therapeutic targets. A recent example is the development of the CRISPR-based genome-targeting tools that carry the potential for genome editing, gene regulation, epigenetic modulation, chromatin manipulation, and live cell chromatin imaging. This fundamental advance, which promises to revolutionize not only the therapeutic approach of genetic disorders but also biological research itself, would not have been possible without a robust discovery science infrastructure.

Whereas the importance of computational science with “big data” is increasingly recognized, some may argue that in the field of critical care medicine, basic science has *not* really helped scientific progress that much. The perception has been voiced that it is a waste of time and money [2], that only a minority of experimental studies have been translated into clinical trials, and none as yet yielded a useful therapy for the critically ill [3]. We would disagree with this view. An example where basic and translational science have led to improvements in critical care is in ventilatory support. Advancements in our understanding of the pathophysiology of ARDS in cellular and animal models have led to intervention trials that have improved outcomes from mechanical ventilation, including lung protective ventilation and extracorporeal support. In fact, a long list of important interventions commonly applied in critical care were also developed through basic science, such as dialysis, antibiotics, insulin, vaccines, pacemakers, hemodynamic monitoring, as well as use of diagnostics such as PCR and imaging techniques.

Another example of a field in which basic and translational science is helping us, is stratification. Knowledge of fundamental biology enabled the re-analysis of intervention trials in hitherto undifferentiated sepsis and ARDS cohorts by stratification of patients into specific populations. Results suggest that patients with the same clinical syndrome but with a different underlying genomic/transcriptomic, and/or immunologic/coagulation host response, have a differential chance to respond to a specific intervention [4, 5]. This development is an example of how advances in molecular biology, genomic sciences, and computational methods help to take a personalized approach to sepsis and ARDS treatments that hold real promise for the finding of targeted therapies. Basic science has enabled us to move into the era of personalized medicine. Such a development was unimaginable even a decade ago.

Despite these successes for basic and translational medicine in critical care, there is lack of success in discovering direct “pharmacologic” therapies for the critically ill. However, this does not mean that basic/translational science in critical care has “failed.” A fundamental aspect of discovery science is that outcome is uncertain and disappointing results are to be expected, as discovery science usually is a complex process and does not follow a linear cause and effect pattern [6]. This may apply in particular to critical care medicine in which disease syndromes are complex and multi-factorial. Consequently, efforts to generate new therapies must continue to be driven by advances in our understanding of the underlying pathophysiology.

What happens if the fundamental understanding of the pathophysiology is incomplete and a more or less “pragmatic” approach is taken in clinical trial design, may be exemplified by sepsis research. Despite numerous trials in which thousands of patients have been included at the expense of millions of dollars, there is no specific therapy for sepsis. An insufficient understanding of this syndrome is at least one of the explanations. In line with this, critical care societies have called for research aimed at understanding the fundamental pathogenesis of sepsis [7]. Without this deep knowledge, researchers choose interventions that appear logical based on their experience, which are then tested in preclinical models that poorly predict efficacy in humans, yielding the development of a drug that after testing in costly clinical trials carried out in undifferentiated populations, that is found to be ineffective.

Despite the importance of discovery science in developing new therapies, in recent years, there has been a significant decline in the number of basic and translational science manuscripts that are published in medical journals with a high impact factor [8]. This decline is manifest across a broad range of medical specialties, including a number of top ranked journals in critical care. Why is this happening? One explanation may be that many physicians are less interested in questions that seem unlikely to directly change their clinical practice. A second issue is that clinically focused manuscripts are more often cited, which improves the impact factor of a journal [8]. Thereby, the preference of editors may move away from publishing basic science research. Consequently, the content of medical journals is changing, with more attention being given to paramedical issues such as medical education, ethics, and clinical epidemiology, with less focus on preclinical research. Some higher impact critical care journals publish little if any preclinical or translational research.

As a consequence, medical trainees may perceive basic and translational research as less relevant to clinical care because it is not published in journals with a high impact factor [9]. The effect is that trainees have less understanding and therefore less interest in the pathophysiology of disease and in mechanisms of therapy. This may affect their clinical practice. The use of protocols has improved our care but may also reduce curiosity and give students a false sense of security (“we are already doing the right thing”).

Another consequence of a declining interest in basic and translational science may be that the opportunities for obtaining funding for this type of research are declining. Overall, governments and foundation sponsors in the Western part of the world spent less money on medical research. Also, the current pattern of investment in medical research is moving away from basic science [10], as funding calls increasingly focus on “direct” clinical impact. Not only governments, also pharmaceutical and biotechnology companies focus on supporting late-stage translational studies and on clinical trials, with a concomitant diminished discovery-level investment. Lastly, few plenary sessions at our critical care conferences concern basic science. Ultimately, a self-fulfilling prophecy may occur: researchers in basic science are regarded as less successful and are no longer a role model for trainees, leading to fewer physicians pursuing a combined career as both a basic/translational scientist and a medical practitioner. This in turn will lead to declining output of basic medical publications.

If basic science disappears from critical care medicine, the absence of early translational components that feed the critical care research “pipeline” would greatly diminish the likelihood of developing therapies for critical illnesses, at a time in which new treatments are urgently needed, given our aging population. We continue to need basic science for fundamental questions that remain in critical care medicine, such as the pathophysiology and treatment of organ failure.

How do we increase the likelihood of successful basic/translational science going forward? We suggest that many current preclinical models in which hypotheses are tested, do not adequately replicate the clinical condition, and need to be improved [11]. Suggestions to do so are listed in Table 1. Promising therapeutic discoveries should be translated through a series of models that more closely reflect the clinical conditions of ICU patients, including multiple hit models that combine injuries with ventilation and interventions, with an appropriate follow-up time for complications to develop and ideally models that include underlying chronic comorbidities that can represent the context of regeneration and frailty [11, 12]. Preclinical models should also consider a standardization and reproducibility context [13–15]. Preclinical studies need to be of the highest quality in design, with blinding, random allocation, and with sample sizes that are sufficient to assess outcomes that have clinical relevance in humans [13–16]. Multi-center preclinical studies demonstrating therapeutic efficacy may further enhance confidence in preclinical research findings. Addressing translational questions such as optimizing dosage regimens may be possible in larger animal models. In addition to animal models, research in organoids provides for excellent opportunities to study the mechanisms of organ failure.

**Table 1:** Suggestions to improve translational study design in critical care

Improving preclinical models	Use of multiple hits
	Incorporate frailty
	Extended duration of models
	Multi-center approach
	Use of randomization
	Bench to bedside communication of phenotype discovery
Improving clinical trials	Mechanistic substudies
	Bedside to bench communication of phenotype discovery
	Testing of pharmacologic strategies in biologically homogenous patient subsets with activation of relevant pathways

The testing of promising potential strategies in these more complex models and incorporating aspects of clinical trial design to enhance rigour may increase later translational success rates. This more complex, longer, and more expensive translational pathway would first need to be validated and proven to be more effective than current approaches. It would also require a change in thinking by preclinical researchers (and by publishers), whereby multi-centric preclinical studies are seen to constitute a valuable contribution, much like participation by centers in randomized clinical trials is currently viewed. Support from funding agencies to conduct these larger preclinical research studies, with their likely substantially greater costs, is also needed. Vice versa, clinical trials increasingly contain an exploratory arm including laboratory science aimed at exploring mechanisms of efficacy. This approach provides for an excellent opportunity for a physician-scientist to learn about the challenges of clinical trial design as well as the translational aspects of science.

To work on the fundamental research questions in critical care, we need critical care physicians interested in basic and translational science, and we should not leave basic science solely to non-clinician scientists [1]. Caring for the critically ill puts critical care physicians in a unique position. Most researchers have a high degree of specialization and may think from the perspective of a single organ, or from the perspective of a single failing biomedical pathway. In contrast, the essence of critical care is that a dysregulated host immune response affects all organs. The critical care physician is trained to take such a holistic systems-based approach. Also, physiology underpins critical care medicine and has helped establish critical care as a specialty in its own right. The intensivist/investigator is best suited to translate physiologic observations from the bedside to the bench.

Besides improvements in models and trial design, we suggest the following solutions to ensure a high level of discovery science in critical care. ICU curricula should reflect the fact that pathophysiology is at the heart of clinical medicine. Trainees who are interested in becoming investigators should be encouraged to pursue translational science research, by establishing mentorship events with scientists and by offering rotations to different laboratories and supervisors. This mentoring requires a new organizational model with specific expertise and dedication by senior faculty members with the aim to integrate basic science work in a clinical background. For the candidate physician-scientist, combining clinical work with research requires an extension of training. Academic institutions should be encouraged to develop a strategy combining these aspects. We need to work with critical care medical journals to emphasize the value of basic science by inviting commentaries on the clinical implications of current advances and by increasing publication of basic and translational science. Lastly, continuous investments are required. New funding sources are needed, which may include crowd funding, or public-private risk sharing collaborations. We need to put effort in helping society understand what critical care medicine entails and which research questions still need to be answered, using social media networks.

## Conclusion

In conclusion, the decline in interest in the role of basic and translational science in discovery of therapies for the critically ill is a cause for concern. We continue to need preclinical models in critical care in which pharmacologic, cell, or gene therapies scan be tested. Basic and translational research findings constitute an opportunity for predictive enrichment of clinical trials and may help to design future trials more efficiently. We call for efforts to narrow the distance between basic, translational, and clinical researchers, moving to larger and multidisciplinary teams to deal with bedside challenges as a common objective. We also propose ways to improve critical care research and increase the profile of basic and translational researchers within the critical care medicine community. This includes support of critical care residents and fellows to perform and publish biomedical research, thereby generating patient-oriented physician-scientists capable of forging links between fundamental biology discoveries and critical care medicine.

## Abbreviations

ARDS: Acute respiratory distress syndrome
CRISPR: Clustered regulatory interspaced short palindromic repeats
PCR: Polymerase chain reaction
ICU: Intensive care unit
